# Electrode Setup for Electromyography-Based Silent Speech Interfaces: A Pilot Study

**DOI:** 10.3390/s25030781

**Published:** 2025-01-28

**Authors:** Inge Salomons, Eder del Blanco, Eva Navas, Inma Hernáez

**Affiliations:** HiTZ Basque Center for Language Technology, University of the Basque Country, Ingeniero Torres Quevedo Plaza, 1, 48013 Bilbao, Spain; eva.navas@ehu.eus (E.N.); inma.hernaez@ehu.eus (I.H.)

**Keywords:** electromyography, silent speech recognition, phone classification

## Abstract

This paper describes a series of pilot experiments developed to define the electrode setup in order to record a novel parallel electromyography (EMG)–audio database. The main purpose of the database is to provide data useful for the development of an EMG-based silent speech interface for Spanish laryngectomized speakers. Motivated by the scarcity of information in related studies regarding this important decision-making process, we decided to carry out a set of experiments with multiple recording sessions and different setups. We included different electrode types (paired and concentric) and locations targeting different muscles in the face and neck involved in the speech production process. We then analyzed the results obtained in a phone classification task using frame-based phone accuracy. The final setup consists of eight channels with bipolar single-electrode pairs targeting eight specific muscles crucial for capturing speech-related information: the digastric, the depressor anguli oris, the risorius, the levator labii superioris, the masseter, the zygomaticus major, the depressor labii superioris, and the stylohyoid. This setup has been used for the final recordings in the database. By providing insight into the electrode setups that were used in related studies and the optimal setup that resulted from this pilot study, we hope that this research will help future researchers in the field in determining their experimental setup.

## 1. Introduction

Speech production is a significant part of human communication and is a complex process consisting of several components working together. It involves transforming a sound source generated by air from the lungs into phones (speech sounds) through articulation by facial, mouth, and tongue muscles. However, for people who have undergone a total laryngectomy, the production of laryngeal speech is no longer possible because they have had their vocal cords removed. The vocal cords are essential for speech production since they add vibration to voiced sounds and determine the pitch and volume of the resulting sound. To help laryngectomized speakers communicate, an alternative to laryngeal speech is required. Several alaryngeal speech methods exist, namely esophageal speech (when air is swallowed and released again making use of vibrating tissue in the esophagus), the use of an electrolarynx (which is an electric device held to the throat to create artificial vibration), or having a tracheoesophageal voice prosthesis following a medical procedure. However, these methods present large disadvantages compared to laryngeal speech, as they are harder to understand by others, and a large part of the speaker’s voice identity is lost [1,2,3,4].

This calls for the development of a silent speech interface (SSI), which is a technological application that outputs a synthetic voice, preferably adapted to the speaker’s original voice, and which is derived from biosignals taken from the brain, muscles, lips, or tongue [5,6,7].

To capture muscle activity, a technique called electromyography (EMG) is used [8]. EMG means “recording (*graphy*) of electricity (*electro*) of the muscle (*myo*)”. A muscle contracts due to electrical impulses activated by signals from the brain and relaxes when there is a lack of those impulses. These electrical impulses can be recorded by an EMG device through electrodes inserted in the muscle (invasive EMG; iEMG) or attached to the skin (surface EMG; sEMG). The resulting EMG signal represents the presence and absence of electricity in the muscle. We have selected sEMG due to its non-invasive nature. Some studies have based their SSI on more invasive methods, such as Permanent Magnet Articulography [9,10], which requires placing magnets on the tongue, as well as on the lips. This method offers the advantage of capturing tongue movements, which are not directly measurable with sEMG, but it is more uncomfortable for the user.

As part of the ReSSInt project [11,12], we wanted to create an EMG and speech database that can be used to develop an SSI for laryngectomized Spanish speakers. For this, it is essential to determine the optimal electrode setup. Decisions have to be made regarding the electrode type, number, and locations while considering practicality and speaker comfort.

There are two ways to acquire EMG signals, namely in monopolar or differential configuration. For monopolar acquisition, a reference electrode is required, which is placed in a location where no activity related to the muscle activity is expected, for example, on the earlobe. Then, the signal from the reference electrode is subtracted from the raw signal from the single monopolar electrode on the target muscle. Differential acquisition means that the difference between the signals acquired in two points is measured. This can be performed using bipolar electrodes (made up of a pair of single electrodes) or an array of at least two electrodes. Two measuring points form one channel, whether that is between two bipolar electrodes, between the reference and a monopolar electrode, or between two electrodes in an array.

Over the years, several electrode setups have been used to acquire EMG from facial muscles, differing in type (monopolar, bipolar, array electrodes, or a combination of them), shape (circular or rectangular), number of channels (ranging from one to more than 100), and locations of the electrodes.

Table 1 provides an overview of previous studies and the electrode setups used. The table is divided into four sections, corresponding to four approaches used to select the electrode locations.

The first approach (section 1) is targeting specific muscles. In a series of studies by different research groups [13,14,15,16,17,18,19,20], a group of five muscles was targeted, namely the levator anguli oris (LAO), the zygomaticus major (ZYG), the platysma (PLT), the depressor anguli oris (DAO), and the anterior belly of the digastric (ABD), i.e., the superficial muscle most related to the tongue. In other studies, different muscles such as the buccinator (BUC), orbicularis oris (OBO), mentalis (MNT), levator labii superioris (LLS), mylohyoid (MLH), sternocleidomastoid (SCM), or the risorius (RIS), i.e., the laughing muscle, were targeted [21,22,23,24,25,26].

In the second approach (section 2), no specific muscles are targeted, but anatomical regions are targeted. A series of studies using this approach by a group of the same researchers includes [27,28,29,30,31,32]. More recently, the same approach was used by Gaddy and Klein [33,34,35].

The third approach (section 3) is a high-density electrode setup without targeting a particular muscle or anatomical region, either using electrode arrays [19,20,36] or all single electrodes [37,38,39,40,41].

A fourth approach (section 4) was proposed recently by [42] in which they used arrays to select eight electrodes to target specific muscles.

From the four approaches, we believe that the one where specific muscles are targeted is the most accurate for the task of predicting speech from facial muscles, considering the physiology of muscles and the muscular anatomy of the face. First of all, while electrode arrays might be suitable for large muscles, we believe it is not the best approach when considering facial muscles. The arrays are rigid and therefore difficult to adjust to the movement of the muscles while speaking. Furthermore, knowing that activation potentials travel along the length of a muscle, longitudinal acquisition of the target muscle is required. However, most facial muscles are long and narrow and close to each other, so using an array (or a high-density setup with single electrodes) increases the risk of cross-talk, which is the interference of muscle activity from surrounding or underlying muscles in the signal of the target muscle.

**Figure A1:**
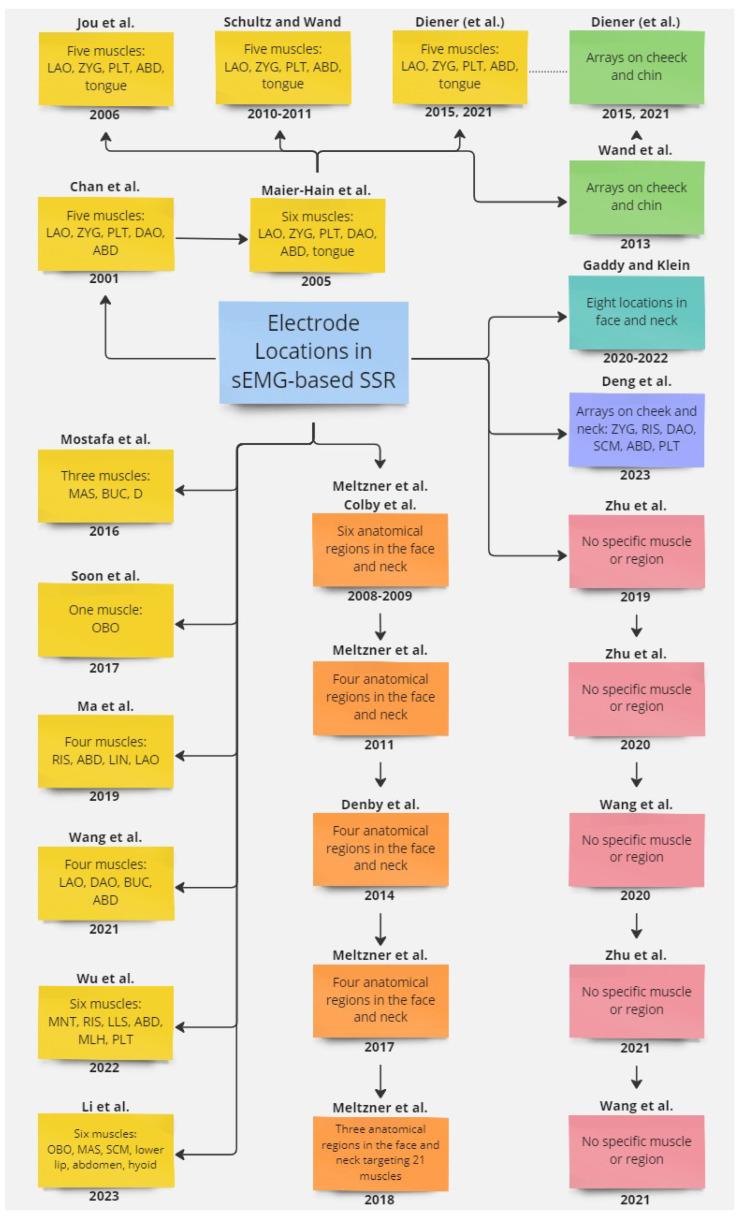
Diagram of studies related to electrode locations used in EMG-based speech recognition.

Figure A1 in Appendix A shows a diagram of the studies and how they are connected. Here you can see that, as is normal in academic research, often one study is a continuation of a previous study by a group of the same authors. However, we realized that the studies that follow the approach that we are interested in (see section one of Table 1) go back to one study [13] or do not provide a systematic approach. Therefore we wanted to conduct a pilot study to find the optimal electrode setup before recording the database. This paper shows the results of that pilot study, comparing electrode types and locations.

Regarding the distinction between monopolar and bipolar EMG acquisition configuration, the studies found [8,43,44,45] are not very conclusive and not focused on small muscles such as muscles of the face. However, we were strongly advised by the equipment provider in favor of bipolar configuration, which we adopted. Bipolar acquisition ensures that there are two measuring points for the same muscle and (if placed correctly) activity from a non-target muscle can be canceled out. We compared two types of bipolar electrodes, namely concentric electrodes and pairs of single electrodes. The results of this experiment are included in this paper. In addition, we tried cup electrodes that are usually used for the scalp due to their smaller size, but they appeared to be too impractical to be used in the face. The main problem was that these electrodes would not stay in place as a result of movements in the face and the weight of the cables.

To select the locations of the electrodes, we targeted 14 muscles in the face and neck of one participant and used the results of per-channel phone classification experiments to discard the least useful channels, resulting in an eight-channel setup targeting five muscles in the face and three in the neck.

This paper is organized as follows. In the next section, the materials and methodology of the pilot study are described in more detail, namely how the data are collected (Section 2.1) and processed (Section 2.2), and the experimental part (Section 2.3) of the classification tasks. Then, the results of the electrode type comparison (Section 3.1) and the channel selection (Section 3.2) are summarized. Finally, we provide a discussion and conclusion (Section 4).

**Table 1:** Electrode number, type, and locations in previous studies. Each of the sections lists studies with a similar approach, which are described in the Introduction. Grouped studies used the same setup.

Reference	Electrode Number and Type	Locations
Chan et al. (2001, 2002) [13,14]	5 pairs	LAO, ZYG, PLT, DAO, ABD
Maier-Hein et al. (2005) [15]	7 pairs (3 bipolar, 4 monopolar)	LAO, ZYG, PLT, DAO, ABD, Tongue
Jou et al. (2006) [16]	6 pairs (2 bipolar, 4 monopolar)	LAO, ZYG, PLT, ABD, Tongue
Schultz and Wand (2010) [17]; Wand and Schultz (2011) [18]	5 pairs (2 bipolar, 3 monopolar)	LAO, ZYG, PLT, ABD, Tongue
Diener et al. (2015) [19]; Diener (2021) [20]	5 pairs (2 bipolar, 3 monopolar)	LAO, ZYG, PLT, ABD, Tongue
Mostafa et al. (2016) [21]	3 electrodes	MAS, BUC, Depressor
Soon et al. (2017) [22]	1 pair	OBO
Ma et al. (2019) [23]	2 monopolar electrodes, 2 bipolar pairs	RIS, ABD, LIN, LAO
Wang et al. (2021) [24]	4 pairs	LAO, DAO, BUC, ABD
Wu et al. (2022) [25]	6 pairs	MNT, RIS, LLS, ABD, MLH, PLT
Li et al. (2023) [26]	6 tripolar	OBO, MAS, lower lip muscle, bi-abdominal anterior abdomen, inferior lateral muscle of the hyoid bone, SCM
Meltzner et al. (2008) [27]; Colby et al. (2009) [28]	11 bipolar bars	supralabial, labial, sublabial, submental neck, midline neck, lateral neck
Meltzner et al. (2011) [29]	8 single-differential bars	submental neck, ventromedial neck, supralabial face, infralabial face
Deng et al. (2014) [30]	4 sensors	above and below the oral commissure, submental surface, ventral neck surface
Meltzner et al. (2017) [31]	8 differential bars	submental, ventromedial, supralabial, infralabial
Meltzner et al. (2018) [32]	11 sensors	submental region, ventral neck, face
Gaddy and Klein (2020, 2021) [33,34]; Gaddy (2022) [35]	8 monopolar electrodes	left cheek just above mouth, left corner of chin, below chin back 3 cm, throat 3 cm left from Adam’s apple, mid-jaw right, right cheek just below mouth, right cheek 2 cm from nose, back of right cheek; 4 cm in front of ear
Wand et al. (2013) [36]	two 1 × 8 strips	cheek, chin
Wand et al. (2013) [36]; Diener et al. (2015) [19]; Diener (2021) [20]	4 × 8 grid, 1 × 8 strip	cheek, chin
Zhu et al. (2019, 2020, 2021) [37,38,41]; Wang et al. (2020, 2021) [39,40]	120 high-density electrodes	cheeks, neck
Deng et al. (2023) [42]	8 electrodes within two 32-channel arrays	ZYG, RIS, DAO, SCM, ABD, PLT

## 2. Materials and Methods

This section describes the methodological part of this study. First, we explain how we collected the data, the materials we used, and the resulting pilot database (Section 2.1). Then, we describe how we processed the signals and extracted features from them (Section 2.2). Lastly, we explain the experiments we performed and the models we used (Section 2.3) and how we evaluated the outcomes of these experiments (Section 2.4).

### 2.1. Data Collection

As described in Section 1, when preparing the data acquisition method for the ReSSInt database, a pilot experiment was conducted to find the most optimal electrode setup in terms of electrode type, size, location, and number. For this, we collected data in three sessions from one male native Spanish speaker. Each session was designed with a different goal in mind and served as data for different experiments (see Section 2.3). Table 2 provides an overview of the electrode setups per session, specifically the muscles that were targeted in each session. We placed the electrodes in the middle of the muscle. In the case of the electrode pairs, the electrodes were placed next to each other in the direction of the muscle fiber. As a reference, we used images of the respective muscles from www.learnmuscles.com (accessed on 15 November 2024) and the 3D anatomy visualizer on www.zygotebody.com (accessed on 15 November 2024).

**Figure 1:**
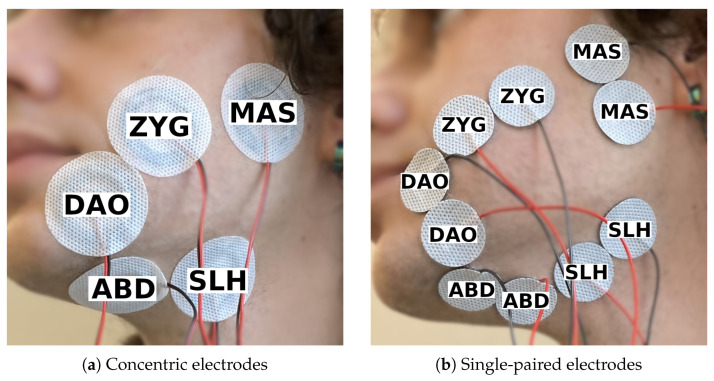
Electrode setup for session 1, made up of five channels targeting the same set of five muscles but using two different types of electrodes.

In terms of electrode type and size, we compared bipolar concentric electrodes (Figure 1a) to bipolar single-paired electrodes (Figure 1b), referred to as Session 1. On the one hand, the positions of the two electrodes in a concentric electrode are fixed, which could help reduce inter-session variability. On the other hand, a concentric electrode has a larger diameter (40 mm) than a single electrode (24 mm), which could result in more cross-talk. There was no inter-electrode distance (IED) between the two bipolar electrodes. The inner diameter of the concentric electrode is 10 mm and the outer diameter is 31 mm.

The participant recorded 250 phonemically balanced short sentences taken from the Sharvard Corpus [46], once with each electrode setup, which consisted of five channels on the left side of the face.

**Figure 2:**
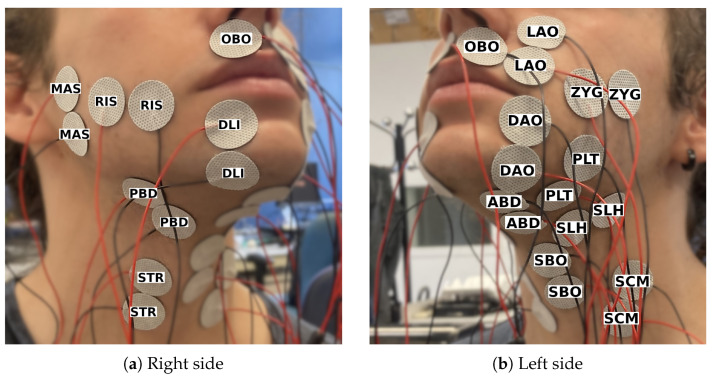
Electrode setup for session 2, consisting of 14 channels targeting a different muscle each.

To see which muscles were most significant, we performed a session (Session 2) in which we placed 14 single-electrode pairs in an attempt to target 14 superficial muscles in the lower face, chin, and neck area (Figure 2). The initial plan was to make the setup symmetrical, but during the electrode placement, it turned out that the 14 channels had to be divided over both sides of the face due to lack of space, resulting in an asymmetrical setup. The participant recorded 105 consonant–vowel (CV) combinations, three times in a row. Each of the 21 Spanish consonants was paired once with each of the five vowels in Spanish, resulting in 105 combinations. Context was added to each combination in the format *ata*[*C*][*V*]*tato* control for co-articulation. The participant could take a short rest whenever they wanted to but preferred not to.

**Figure 3:**
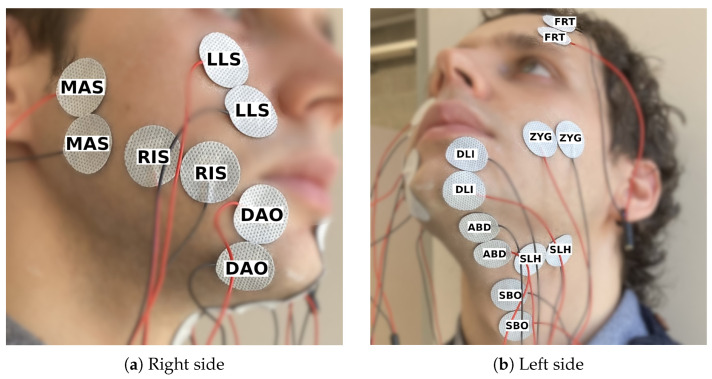
Electrode setup for session 3, consisting of 10 channels targeting a different muscle each.

After analyzing and comparing the 14 channels of session 2 (Section 3.2), we recorded another session (Session 3) to finalize the electrode setup. The 250 sentences from the Sharvard Corpus were recorded two times. See Figure 3 for the electrode setup.

In a sound-proof room, we collected the data with the following equipment: a Quattrocento bio-electrical amplifier from OT Bioelettronica to obtain the EMG signals (with a sampling frequency of 2048 Hz) and a Neumann TLM103 microphone to record the voice (with a sampling frequency of 16 kHz). We used a so-called silent computer to record the data, which uses surface heat dissipation instead of internal fans to reduce both acoustic and electrical noise.

To ensure that the EMG signals and the audio are well aligned, a synchronization signal is shared between the bio-electrical amplifier and the sound device. The synchronization signal is raised by the speaker through the recording interface when they start the recording of each utterance, and it is lowered when they finish the recording. The electrical bio-amplifier creates the synchronization signal and it is saved together with the EMG signals as an additional channel. At the same time, it is outputted through an analog auxiliary output, which is introduced in one of the channels of the sound interface. The stereo audio signals contain the speech signal in the left channel and the synchronization signal in the right channel. Then, both EMG signals and audio signals are cut using the synchronization signal to ensure that they both belong exactly to the same time interval.

The 26 phone classes present in the CV combinations are B, D, G, J, T, a, b, d, e, f, g, i, jj, k, l, m, n, o, p, r, rr, s, t, tS, u, and x. These phones are based on the Speech Assessment Methods Phonetic Alphabet (SAMPA) for Spanish (https://www.phon.ucl.ac.uk/home/sampa/spanish.htm, accessed on 15 November 2024). The number of phone classes for the sentence set is slightly higher because we created the dictionary automatically, resulting in some additional phones. In total, there are 29 phones and the additional ones are L, j, w. The latter two are the Spanish semi-vowels, which we left out of the CV combinations since they are neither consonants nor vowels. The L is not commonly used in Castillian Spanish, which is why we did not consider it when creating the CV combination dictionary manually. We split the data of each session into 80% for training and 20% for testing. We made sure that the balance of CV combinations was similar for the training and test sets. For the 250 sentences recorded in sessions 1 and 3, we assigned the last 20% of the sentences to the test set. See Table 3 for the amount of data in time for each subset of each session.

### 2.2. Signal Processing and Feature Extraction

To perform the phone classification experiments, the raw EMG and audio signals needed to be processed and parameterized.

First, both audio and EMG signals were cut using the synchronization signal. Subsequently, each audio signal was automatically aligned with its phonetic labels using the Montreal Forced Aligner [47].

Then, we parameterized the EMG signals by calculating a set of time-domain (TD) features. These features have been widely used in works related to EMG signals applied to speech recognition or generation [16,33,48,49,50]. Consistent with this established body of work, we have applied the same parameterization procedures described in the literature. Although other options could have been considered, the focus of this study is not on developing or evaluating new parameterization methods but rather on exploring the impact of sensor placement on performance. We opted for the use of the same parameters and parameterization methods to simplify future results comparisons. The initial step involves the removal of direct-current offsets from each individual EMG signal corresponding to the duration of each utterance delimited by the synchronization signal, from a single channel, followed by normalization through division of each signal by its maximum absolute value.

To obtain the TD features, we first separated the EMG signal (x[n]) into a low-frequency signal (w[n]) and a high-frequency signal (p[n]). The low-frequency signal was obtained by calculating a double average of x[n] using a nine-point window. The calculation can be expressed as:

(1) $$w\left[n\right]=\frac{1}{9}\sum_{k=-4}^{4}v\left[n+k\right],\;\mathrm{where}\;v\left[n\right]=\frac{1}{9}\sum_{k=-4}^{4}x\left[n+k\right]$$

The impulse response of a double-pass moving average filter with *N* points is the triangular function defined by:

(2) $$h_{w}\left[n\right]=\left\{\begin{array}{cc} \frac{1}{N}\left(1-\frac{\left|n\right|}{N}\right) & \mathrm{if}\;|n|\leq N\\ 0 & \mathrm{otherwise} \end{array}\right.$$

where *N* is set to 9 for the filter defined previously.

The frequency response of this filter corresponds to a squared sinc function, characterized by zero-crossing points at frequencies $f=f_{s}\cdot \frac{k}{N}$, where *k* is an integer different from zero, $f_{s}$ denotes the sampling frequency (set to 2048 Hz), and *N* equals 9. The filter’s bandwidth, defined as the frequency range where power attenuation remains below −3 dB (equivalent to an amplitude ratio of 0.707), is constrained to frequencies under 73.01 Hz. The frequency response is mathematically expressed as:

(3) $$H_{w}\left(f\right)=\left\{\begin{array}{cc} 1 & \mathrm{if}\;f=0\\ {\left(\frac{\sin(\pi fN)}{N\sin(\pi f)}\right)}^{2} & \mathrm{otherwise} \end{array}\right.$$

**Figure 4:**
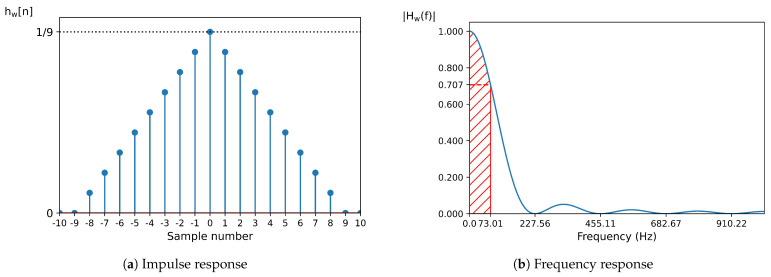
Impulse response (**a**) and frequency response (**b**) of the double-pass moving average filter. In the frequency response, the frequencies marked in the x-axis correspond to the zero-crossing points. The filter’s bandwidth, indicated in red, corresponds to the frequency range where the amplitude remains above 0.707.

The graphic representation of the impulse and frequency response can be observed in Figure 4.

The high-frequency signal, p[n], was obtained by subtracting w[n] from x[n]. This can be represented as:

(4) p[n]=x[n]−w[n]

In addition, a rectified version of the high-frequency signal, r[n], was calculated as follows:

(5) $$r\left[n\right]=\left\{\begin{array}{c} p[n],\;\mathrm{if}\;p[n]\geq 0\\ -p[n]\;\mathrm{if}\;p[n]<0 \end{array}\right.$$

With the low-frequency signal (w[n]), high-frequency signal (p[n]), and rectified high-frequency signal (r[n]) obtained, we computed the set of five TD features for each frame, using a window with a duration of 25 ms and a frame shift of 5 ms. For w[n] and r[n], the frame-based power ($P_{w}$ and $P_{r}$) and the frame-based time-domain mean ($\bar{w}$ and $\bar{r}$) are calculated, and for r[n] the frame-based zero-crossing rate (*z*) is calculated. These features are defined as:

(6) $$TD0=[\bar{w},\bar{r},P_{w},P_{r},z]$$

where:

(7) $$\bar{w}=\frac{1}{N}\sum_{n=0}^{N-1}w\left[n\right],\;\bar{r}=\frac{1}{N}\sum_{n=0}^{N-1}r\left[n\right]$$

(8) $$P_{w}=\frac{1}{N}\sum_{n=0}^{N-1}{\left|w\left[n\right]\right|}^{2},\;P_{r}=\frac{1}{N}\sum_{n=0}^{N-1}{\left|r\left[n\right]\right|}^{2}$$

(9) $$z=\sum_{n=1}^{N-1}g\left(p\left[n\right]p\left[n-1\right]\right),\;\mathrm{where}\;g\left(x\right)=\left\{\begin{array}{c} 1\;\mathrm{if}\;x<0\\ 0\;\mathrm{if}\;x\geq 0 \end{array}\right.$$

where *N* denotes the number of samples in x[n]. To incorporate temporal context into the features, a stacking filter was used to concatenate the features of 2k+1 adjacent frames, where *k* represents the width of the stacking filter. We selected k=15, resulting in a total of 31 frames being combined, with the analyzed frame in the center. The stacked TD0 vectors from all channels were then combined into a single array, which served as the input for the classifier. The length of the parameter vector assigned to each frame can be calculated as:

(10) M·5·(2k+1)

where *M* represents the number of channels.

To reduce the dimension of the parameter vector, we applied linear discriminant analysis (LDA) [51], as performed in [18,48], to obtain a set of LDA features. The number of features is equal to the number of classes present in the data (phone labels) minus 1, which is the maximum allowed number of features in LDA reduction. In the case of the VC dataset, this resulted in 25 features, and in the case of the sentences, this resulted in 28 features.

### 2.3. Experiments

With the three experiments we performed, we had two goals.

The first goal was to find out which type of bipolar electrodes would yield the highest accuracy. We used the data from session 1 and performed a phone classification task using the signals of all five channels, one time with the signals from the concentric electrodes and another time with the single paired electrodes.

The second goal was to select the optimal set of electrodes regarding their number and locations. For this, we performed two experiments with the data from sessions 2 and 3. To assess the amount of information provided by each muscle, a phone classification experiment was performed using the signals from one single channel each time. The muscles that achieved the highest accuracy were considered to contain the most useful information to perform the task.

Since the size of the data set used in each experiment is limited to only one session, we did not want to base our conclusions on one classifier only and decided to compare three classifiers. The first is a Gaussian mixture model (GMM), which has been used in phone classification experiments before [18]. The second is a bagging classifier with decision trees (DTs) as estimators, which we thought appropriate for the small data size. The third is a feed-forward neural network (NN), as in [52], which we wanted to include since NNs are the most standard type of machine learning model used in recent years.

The maximum number of components in the GMMs was 29, which is the number of classes minus 1. Starting with 1 component, it continued adding components until the Bayesian Information Criterion of the new model was higher than the last model’s BIC.

The number of decision trees for the DT models was 50 for session 2 and 100 for sessions 1 and 3. The minimum number of samples in the leaf node was set to 5 for session 2 and to 10 for sessions 1 and 3. These parameters were set following a parameter-tuning experiment, in which we tried different combinations of parameter values and chose the one that resulted in the highest validation accuracy.

For the NN, we used one hidden layer with twice the number of features as input nodes and the ReLU activation function. The output layer consisted of as many nodes as there are phone labels and the softmax activation function. It was compiled using the cross-entropy loss function and the Adam optimizer. We used a batch size of 32 and a training size of 25 epochs for the experiments with data from session 2 and a batch size of 64 and a train size of 50 epochs for the experiments with data from sessions 1 and 3. These parameter values were determined after training a classifier for 100 epochs with batch sizes 32, 64, and 128 and choosing the combination at the point when the validation accuracy stopped increasing.

For each model, 5-fold cross-validation was implemented on the train set (which is 80% of the complete data set).

### 2.4. Evaluation

We used the mean frame-based phone accuracy of the five validation sets after 5-fold cross-validation as an evaluation measure to select the electrode locations and type. For session 1, we applied a Wilcoxon Signed-Rank Test to check for statistical differences. We used the test accuracy to evaluate if the final setup was appropriate.

## 3. Results

This section summarizes the results of the experiments. First, the results of the comparison of electrode types (Section 3.1) and then the results that were used to select the channels (Section 3.2) are shown.

### 3.1. Electrode Type

**Figure 5:**
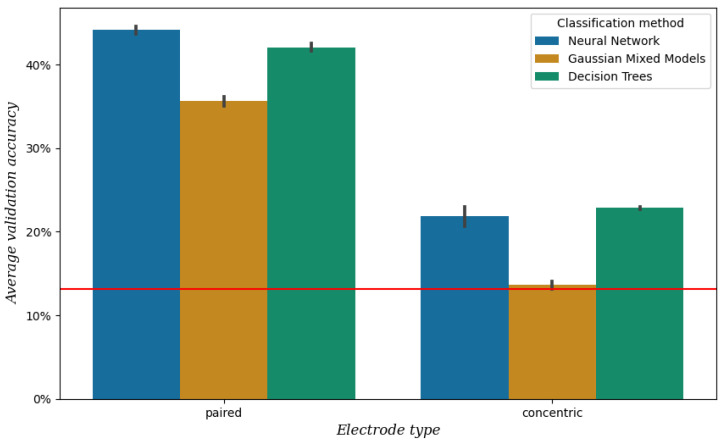
Mean validation accuracy after 5-fold cross-validation per electrode type and classification method, obtained from the data of session 1. The vertical bars represent the confidence intervals, and the red line represents the baseline, which is the mean validation accuracy when always predicting the most frequent class [a].

As described in Section 2.3, we compared two types of electrodes in bipolar configuration: concentric electrodes and pairs of single electrodes. See Figure 5 for the mean validation accuracy obtained from the data of session 1, which is significantly higher when using paired electrodes compared to concentric electrodes (*p* < 0.001). For this reason, we chose the single paired electrodes for our setup.

### 3.2. Channel Selection

**Figure 6:**
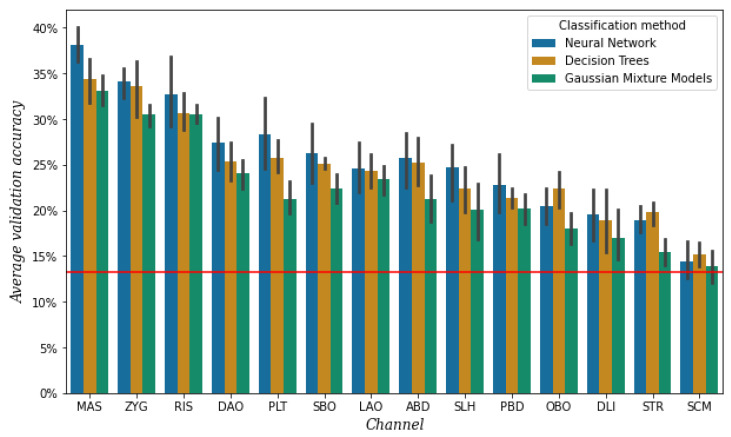
Mean validation accuracy after 5-fold cross-validation per channel and classification method, obtained from the data of session 2. The vertical bars represent the confidence intervals, and the red line represents the baseline, which is the mean validation accuracy when always predicting the most frequent class [e].

To select the most useful channels, we performed a simple phone classification task per channel with the data from session 2, using the results of three classifiers. For detailed information on the classifiers, see Section 2.3. The average validation accuracy per channel and classifier is shown in Figure 6.

It is important to mention that the electrodes of the three channels around the mouth, namely both electrodes of OBO and the top electrodes of DAO and DLI, did not stick as well as the electrodes of the other channels. This was most likely due to the area under the electrodes being curved as a result of lip movement. We had to reattach these electrodes a few times during the session. The OBO channel, as it was affected by sweat and condensation of air coming from the nose as well, was the most problematic.

The highest validation accuracy is achieved by a different classifier for the different channels, as shown by Figure 6. However, for all three classifiers, it appears that both separately and averaged the six channels with the lowest accuracy are SLH, PBD, OBO, DLI, STR, and SCM. As mentioned before, the electrodes of the DLI channel did not always attach well. We repeated the experiment with the data of the three rounds of the sessions separately, and it turned out that DLI channel belonged to the top five of highest accuracy in the first round but decreased with each round. For this reason, we decided not to discard this channel yet.

After discarding channels OBO, STR, and SCM for their low performance, we took another look at the muscular anatomy of the remaining channels. The muscles SLH and PBD are located very close together and perform similarly as well, so we decided to only discard channel PBD and keep SLH, although in practice channel SLH most probably represents information from both muscles. In addition, we realized that the LAO is a very short muscle, but that the LLS is a closely located but longer muscle. So we decided to replace LAO with LLS because longer muscles are easier to target and additionally to avoid the area directly above the lips. Furthermore, we saw that the PLT is a broad sheet of muscle instead of a muscle with a more specific location, making it difficult to know whether the information we are measuring belongs to this muscle. Therefore, we decided to remove the channel corresponding to PLT.

Additionally, we added one new channel for the muscle of the forehead (FRT). The purpose of this channel was to be used as a reference as it was not expected to provide any muscle information related to speech.

Finally, the set of 10 channels included in the next recording session (session 3) was the following: MAS, ZYG, RIS, DAO, SBO, LLS (instead of LAO), ABD, SLH, DLI, and the new one FRT.

**Figure 7:**
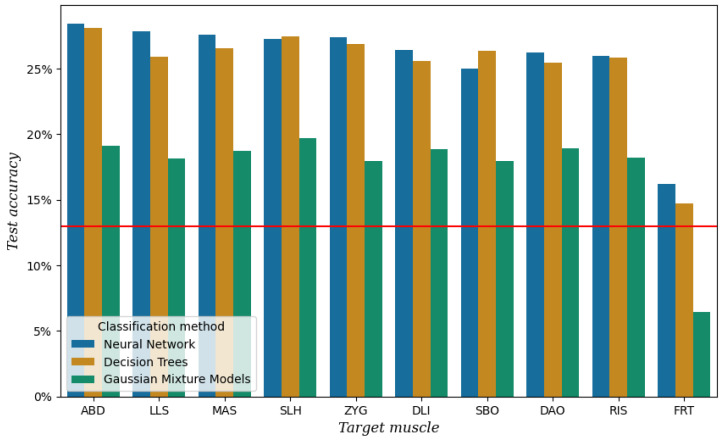
Test accuracy per channel and classification method for the data of session 3. The red line represents the baseline, which is the test accuracy when always predicting the most frequent class [a].

Figure 7 shows the test accuracy per channel and classifier after performing the classification experiment described in Section 2.3 on the data from session 3. It can be seen that the channel with the lowest test accuracy is FRT with a performance similar to baseline. This result provides an extra assurance that the other channels indeed carry some information related to speech production. The highest test accuracy when using all the channels except FRT was achieved with an NN at 48.42%.

For the final setup, we left out FRT for obvious reasons, but SBO as well. This channel is located in the area where the stoma is located in laryngectomized speakers. For studies with a different target group, this muscle might be a valuable addition, but for our study, we realized it was not practical.

The final setup, containing ABD, LLS, MAS, SLH, ZYG, DLI, DAO, and RIS, has been used to record the ReSSInt database. The recordings for this database are still ongoing, and the official database will be released once they have finished.

## 4. Discussion

In this paper, we present a series of pilot experiments we conducted to find the optimal electrode setup for developing a database of EMG and (silent) speech data. Following a common approach in previous studies where individual muscles in the face and neck are targeted [13,14,15,16,17,18,19,20,21,22,23,24,25,26], we initially looked at the contribution of 14 individual muscles in a phone classification task. For the final setup, we decided to include eight bipolar single-electrode pairs targeting one muscle each, of which five are located in the face and three in the neck, in an asymmetrical setup. Out of the eight muscles, six are present in the setups of at least one of the studies mentioned above as well, namely the anterior belly of the digastric (ABD), the depressor anguli oris (DAO), the risorius (RIS), the levator labii superioris (LLS), the masseter (MAS), and the zygomaticus major (ZYG). The levator anguli oris (LAO) is more commonly used instead of LLS, but we chose LLS because it is longer. There are two more muscles that we included, namely the depressor labii superioris (DLI) and the stylohyoid (SLH). As far as we know, these muscles have not been used in previous research; however, they have proven to be valuable in our experiments.

One important limitation of the present study is that the experiments have been performed with only one speaker. As could be expected, and experiments performed with data from the ReSSInt database show, there is variability among speakers and even among sessions in the results [53]. This consideration is of special importance for the case of laryngectomized speakers, whose muscles are notably affected by the surgery and probably by radiotherapy received. However, the channels that we discarded not out of practical reasons had a noticeably lower performance than the channels we selected. We believe that the selected setup could be generalized to other speakers, as the muscles used for articulation are the same regardless of individual variance in the manner of articulation, but that some channels might be more useful than others depending on the speaker. In this sense, in a future study, we are considering increasing the number and variability of subjects under analysis.

Additionally, due to the lack of space on the face of the speaker, we had to place the electrodes asymmetrically, and we assumed that this would not cause any difficulties since the musculature of the face is in theory symmetrical. Multiple studies listed in Table 1 use an asymmetric setup. However, we acknowledge that there is a possibility that the results could have turned out differently if we mirrored the setup and that this has to be researched further.

Our setup consists of eight channels, which is more than the number used in related studies, which varies and includes one [22], three [21], four [23,24], five [13,14,17,18,19,20], six [16,25,26], and seven channels [15]. Exploring optimal configurations for the positions of the sensors is a future line of work. For example, in [54], each sensor was assigned a Phoneme Selectivity index to evaluate how neural patterns are related to specific acoustic features. Similarly, the sequential forward selection (SFS) algorithm has proven effective for selecting the most relevant EMG sensors [41]. This method iteratively identifies optimal channels, adding one channel at a time to maximize classification accuracy in combination with previously selected channels.

With the data we already have from the ReSSInt database, we are currently performing a channel-by-channel analysis across sessions and speakers to better understand the real impact of each channel. Note that for the pilot study experiments described in this paper, we looked at the impact of each channel individually. However, we assume that for the production of each (combination of) sound(s), not one but at least a group of two muscles (channels) are responsible. This assumption is included in the channel-by-channel analysis, and we understand that this can result in a reduction in the number of channels that we have selected now.

In addition, we are working on different tasks such as word classification, EMG-to-text, and EMG-to-speech. Unfortunately, we have found that EMG acquisition is a sensitive technique, meaning that variability between signals occurs, even if the participant, electrode setup, recording environment, and uttered speech are the same. This is why in the database recording sessions we include videos from the face as well, which will provide information from the lip movements in order to improve the interface.

Once the ReSSint database is completed, it will be made publicly available through ELRA (https://catalog.elra.info/en-us/, accessed on 15 November 2024) for research purposes.

## Figures and Tables

**Table 2:** Electrode setups (targeted muscles) for each session in the pilot study plus the final setup that is used for the final database.

Muscle	Session 1	Session 2	Session 3	Final Setup
anterior belly of the digastric (ABD)	X	X	X	X
depressor anguli oris (DAO)	X	X	X	X
depressor labii inferioris (DLI)		X	X	X
frontalis (FRT)			X	
levator anguli oris (LAO)		X		
levator labii superioris (LLS)			X	X
masseter (MAS)	X	X	X	X
orbicularis oris (OBO)		X		
platysma (PLT)		X		
posterior belly of the digastric (PBD)		X		
risorius (RIS)		X	X	X
sternocleidomastoid (SCM)		X		
stylohyoid (SLH)	X	X	X	X
sternothyroid (STR)		X		
superior belly of the omohyoid (SBO)		X	X	
zygomaticus major (ZYG)	X	X	X	X

**Table 3:** Overview of the duration of the training and test data sets (format: mm:ss).

	Electrode Setup	Corpus	Size of Train Set	Size of Test Set
Session 1	5 paired	250 sentences	09:12	02:16
	5 concentric	250 sentences	09:02	02:17
Session 2	14 paired	105 CV x3	01:00	00:15
Session 3	10 paired	250 sentences x2	17:16	04:23

## Data Availability

The data used in this study can be made available upon reasonable request.

## Abbreviations

The following abbreviations are used in this manuscript:

ABD	anterior belly of the digastric
BUC	buccinator
CV	consonant-vowel
DAO	depressor anguli oris
DLI	depressor labii inferioris
DT	decision trees
EMG	electromyography
FRT	frontalis
GMM	Gaussian mixture model
LAO	levator anguli oris
LDA	linear discriminant analysis
LLS	levator labii superioris
MAS	masseter
MLH	mylohyoid
MNT	mentalis
NN	neural network
OBO	orbicularis oris
PBD	posterior belly of the digastric
PLT	platysma
PSD	power spectral density
RIS	risorius
SBO	superior belly of the omohyoid
SCM	sternocleidomastoid
SLH	stylohyoid
SSI	silent speech interface
STR	sternothyroid
TD	time-domain
ZYG	zygomaticus major
